# Feasibility and Safety of Mesocolon Excision with Medical Imaging: A Systematic Review and Meta-Analysis

**DOI:** 10.1155/2023/6198625

**Published:** 2023-02-18

**Authors:** Chengkui Liu, Qiang Zhao, Zhichao Song

**Affiliations:** ^1^Department of Gastrointestinal Surgery, Zibo Central Hospital, Zibo, Shandong Province 255000, China; ^2^Department of Anorectal Surgery, The First Hospital of Zibo City, No. 4 Emei Shandong Road, Zibo City, Shandong Province 255200, China

## Abstract

The development of new technologies based on electronic intelligent images is a very active research and promotion of new technologies in recent years. This article mainly summarizes the basic concept, development, and technology of electronic intelligent imaging technology, as well as the research, promotion, and application of electronic intelligent imaging technology in clinical treatment. It especially emphasizes the practicality and application of electronic intelligent imaging technology in the current clinical operation process and conducts a meta-analysis of the current mesorectal excision, so as to provide more scientific and professional guidance for clinical surgery. The results of the meta-analysis showed that 3291 documents were initially obtained and duplicate documents were deleted by searching for keywords in mesocolon excision. We excluded 2399 subjects and articles whose interventions did not meet the inclusion criteria of this study after reading the title and abstract. Then, we obtained 892 papers that may meet the inclusion criteria through preliminary screening. We further optimized the search strategy based on selection criteria and data integrity filtering principles and finally determined 111 references. 100 articles that did not meet the requirements were excluded, and 11 articles were finally included for meta-analysis. Medical imaging can effectively improve the therapeutic effect of mesocolon excision and reduce the occurrence of complications. Therefore, it is very important to combine medical intelligent images for preoperative evaluation, and the development of the combination of surgical treatment and medical images should not be underestimated in the future.

## 1. Introduction

With the development of medical imaging technology, people combine clinical treatment in different fields with medical imaging more closely [1]. At the same time, the combination of medical imaging and surgical diagnosis and treatment can effectively improve the postoperative rehabilitation effect and quality of life of patients [2]. The purpose of this study is to make statistics on the postoperative complications of patients undergoing mesocolon excision and analyze the treatment differences between laparoscopic and open surgery. We try to help medical personnel better perform mesocolon surgery, so as to better develop medical plans.

Total mesocolectomy (TME) was first proposed by a professor head of the United Kingdom in 1988 and gradually developed into a mature surgical treatment scheme. It is believed that rectal cancer surgery should not only remove the focus but also comprehensively remove the diseased tissues including lymph nodes, so as to improve the rehabilitation effect of patients [3]. With the development and wide application of TME, the surgical effect of rectal cancer has been significantly improved. Like rectal cancer, lymph node metastasis is the first metastasis of colon cancer. A large number of clinical studies have shown that fully resecting regional lymph nodes can significantly improve the prognosis and quality of life of patients [4, 5].

The appearance of laparoscopic surgery has further improved the treatment effect of related surgery. Laparoscopic surgery has been applied in Russia since 1901. China successfully completed the first laparoscopic surgery in 1991. In 1901, Ott, a gynecologist in Petersburg, Russia, made a small incision in the front wall of the abdomen, inserted a speculum into the abdominal cavity, and reflected the light into the abdominal cavity with a head mirror to examine the abdominal cavity. In the same year, German surgeon Kelling inserted a cystoscope into the abdominal cavity of the dog for examination and called this examination laparoscopic endoscopy. In 1938, the Hungarian surgeon Veress introduced an air injection needle, which can safely make pneumothorax; During pneumoperitoneum, the needle tip can be prevented from damaging the viscera under the needle [6]. The idea of making pneumoperitoneum with a compromise safety puncture needle is generally accepted and has been used up to now. Kalk, a German gastroenterologist, invented a lens system with a direct forward squint of 135° and first advocated the use of double trocar puncture needle technology in 1929 [7]. Relevant research found that nearly 1/3 of gynecological operations in the Cedars snai Medical Center in Los Angeles were diagnosed or treated with laparoscopy.

This article reviews the concept, development, research, and application of mesocolon excision and laparoscopy. Meta-analysis was used to analyze the complications of patients after mesocolon excision in China, especially the practical application effect of laparoscopy in surgical treatment. The auxiliary treatment of medical imaging technology can reduce the inflammation of patients to a certain extent. Advanced laparoscopic technology can reduce the external trauma during the operation and improve the operation quality to a certain extent.

In order to analyze and summarize the application of mesocolon excision in clinical practice in China, we searched all relevant nursing disciplines in China National Knowledge Network (CNKI) and web of science. Meta-analysis articles were retrieved from the establishment of the database to August 2022, and the retrieved documents were analyzed. Finally, a total of 892 articles were included in the analysis. By deleting duplicates, a total of 111 articles were included in the analysis, accounting for 12.44% of the total included articles. Through the final screening, a total of 11 articles were obtained. Improve the quality of literature screening by increasing the year of analysis. However, the number of high-quality meta-analysis articles is relatively small. On the one hand, meta-analysis can be widely used in the evaluation of clinical surgical results. On the other hand, Chinese clinical workers should make better use of the best evidence and write high-quality articles.

## 2. Decision Tree Algorithm and Laparoscopic Image Technology

### 2.1. Decision Tree Algorithm

The current sample set is divided into two subsample sets by decision tree algorithm using binary recursive segmentation technology. Generally, according to specific segmentation rules, select specific attribute values as branch points, divide the data set into two subsets and repeat this process [8]. When the current subset satisfies the specified segmentation end rule, the growth of the tree stops. After the decision tree is established, the path from the root to the leaf node corresponds to some corresponding logic and rules.

#### 2.1.1. Growth of Decision Tree

In this article, the sample set divided by the CART algorithm refers to the microarray data. The root node is the sample to be divided in the entire microarray dataset, where each gene is the attribute to be selected. And the bifurcation node of the tree is a selected attribute value, and the leaf nodes of the tree represent the class to which the tester belongs [9]. The growth process of the decision tree is the construction process of the classifier.

*(1) Error Index*. The error index is used to quantify the performance of tree node *d* branch samples from different classification problems. The error index of this classification tree is often called a mixed subfunction [10].

In the classification tree, the most famous mixture degree function is the Gini index. The Gini index is the most popular and most commonly used division rule, and it can be used well for data containing noise [11]. In the CART algorithm, Gini's indicator is often the default division indicator, because its performance is often the best. It can be used as an error indicator to generate a tree, and it is defined as follows:

(1) $$\begin{array}{c} \phi \left(H\right)=-\underset{L\neq 1}{\sum }hKhn=1-\overset{L}{\underset{L=1}{\sum }}hL^{2}\text{.} \end{array}$$

Among them ∑_*L*=1_^*L*^*hL*=1, the collection of microarray data samples to be classified can be regarded as the nodes of a classification tree, and the mixing degree function *φ* (*H*) is used to express the mixing degree index of the tree node *d*, which is

(2) $$\begin{array}{c} M\left(d\right)=\phi \left(h1,h2,hL\right)\text{.} \end{array}$$

When *L* = 2, *M* (*d*) ∈ [0, 1/2], the smaller its value, the more the two types of samples in the tree node *D* sample set tend to be one type of sample, that is, the purer.

*(2) The Rule of Division*. If the mixed order function used to calculate the cost of the node is selected, each time the tree grows, the best branch value that divides the sample in the node into the smallest cost will be found [12]. In the binary tree constructed by the CART algorithm, the amount of change in the degree of mixing caused by the branch is as follows:

(3) $$\begin{array}{c} \Delta M\left(d\right)=M\left(d\right)-hlM\left(dl\right)-hrM\left(dr\right)\text{,} \end{array}$$

*d* is the node that is forking; *M* (*d*) is the degree of mixing of node *d*; *M* (dl) and *M* (dr) are the mixing degree of left and right branch nodes respectively; hl and hr are the percentages of left and right bifurcated samples in node *d*, respectively. For the bifurcation of each inner node *d*, taking the one with the largest amount of change in the degree of mixing among all possible bifurcation modes of *d* [13].

*(3) End of Division*. If one of the following conditions is met, the sample segmentation will be stopped:

  First, when the samples contained in a certain node belong to the same category, it is already the purest, and the segmentation is stopped
  Second, when there are no remaining attributes as the basis for sample division, stop the division
  Third, the user sets the maximum depth of the tree in advance to directly limit the growth of the tree and stop the segmentation

#### 2.1.2. Pruning of Decision Tree

The pruning process of the decision tree aims to find the optimal tree partition and reduce the model overfitting by improving the generalization performance of the decision tree. This is an effective means to ensure that the generated classification tree can match the new data through appropriate training. Therefore, there needs to be a way to stop the growth of the tree at an appropriate time, which is the so-called prepruning method [14]. Among them, one is to set the maximum depth of the tree to directly limit the growth of the tree, and the other is to set the minimum number of records contained in each node. The model stops splitting when the number of records in the node is less than this value [15]. The opposite of prepruning is the postpruning method that allows the tree to grow fully and then prunes it later. For the fully grown tree, selectively pruning upwards to obtain a pruning sequence of a candidate subtree. Then, using an independent test data set or cross-validation method to identify the subtree with the lowest error classification rate from these candidate subtrees as the best subtree [16].

*(1) Definition of Pruning*. Generally, a tree can be represented by *S*, and the subtree whose root node is *s* is represented by *S*_*s*_, then the pruned subtree *S*_*s*3_ is shrunk into an end point *s*3. The pruned tree can be represented as *S* − *S*_*s*3_, and *S* − *S*_*s*3_ ⊂ *S*, using$\bar{S}$ to represent the set of endpoints in the tree *S*, and the number of corresponding endpoints is $\bar{S}$. The definition of tree *S* impurity index is

(4) $$\begin{array}{c} M\left(S\right)=\underset{s\in S}{\sum }M\left(s\right)\text{.} \end{array}$$

*M*(*S*) is the index of mixing degree of tree node *s*.

When pruning a decision tree, it needs to pass a cost complexity test first. The cost complexity test is defined as follows:

(5) $$\begin{array}{c} M_{\delta }\left(S\right)=M\left(S\right)+\delta \left|\bar{S}\right|\text{.} \end{array}$$

In the formula, *M*_*δ*_(*S*) is the linear combination of the cost *M*(*S*) of the tree and its complexity. For each given value of *δ*, the corresponding cost complexity test can always find a minimum subtree *S* (*δ*):

(6) $$\begin{array}{c} M_{\delta }\left(S\left(\delta \right)\right)=\underset{S\subset S\max}{\min M_{\delta }\left(S\right)}\text{.} \end{array}$$

*(2) Principle of Pruning*. The main idea of gradual pruning: assuming that the tree *S* has *N* end points, that is, constantly looking for smaller and smaller tree sequences [17]. *θ* is the penalty cost of each node: a number that starts from 0 and increases. When *θ* = 0, there is no penalty for the nodes of the tree, and the cost complexity is measured by the tree that has not started pruning. When *θ* is increased to a large value, the penalty cost for misclassification can be almost ignored in the cost complexity measure, and the minimum tree obtained is the smallest and simplest tree, that is, there is only one node [18].

The method of finding the next minimum tree of the tree *S* is: for each internal node *n* of the tree *S*, the value of *θ* of the next tree *S*-Ss needs to be obtained, denoted as *θ* s. This value represents the ratio of the change in the error index before and after the pruning of the current tree to the change in the number of endpoints:

(7) $$\begin{array}{c} \theta_{s}=\frac{M\left(s\right)-M\left(S_{s}\right)}{-1}\text{.} \end{array}$$

In the pruning process of the decision tree, multiple candidate tree sequences will be obtained. To choose the optimal scale tree, it can be measured by two methods [19]. The simplest is to use new sample data to split the candidate tree so that it is easy to pick the tree with the smallest error test.

### 2.2. Laparoscope

With the development of modern medical technology, laparoscopy, as an extension of doctors' eyes and hands, has reached the state of “omnipotence” [20]. Laparoscopic treatment can effectively detect abdominal inflammation, ulcers, and benign and malignant tumors. In addition, through laparoscopic adjuvant treatment, the location and scope of organ lesions can be located, and biopsies and color painted specimens can be performed. It can not only effectively diagnose diseases, but also carry out targeted treatment [21].

**Figure 1:**
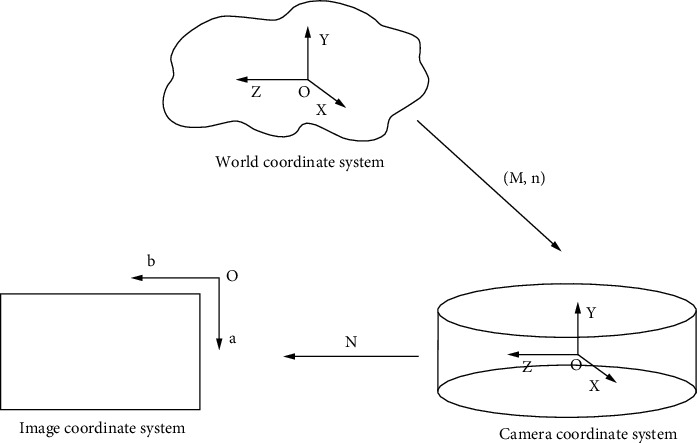
Schematic diagram of perspective projection inverted imaging geometry.

In the process of obtaining the laparoscope, the 3D scene of the objective world needs to be projected onto the 2D image plane of the camera [22], and this projection can be explained by image conversion.  Figure 1 shows a coordinate system related to 3D space scene imaging.

**Figure 2:**
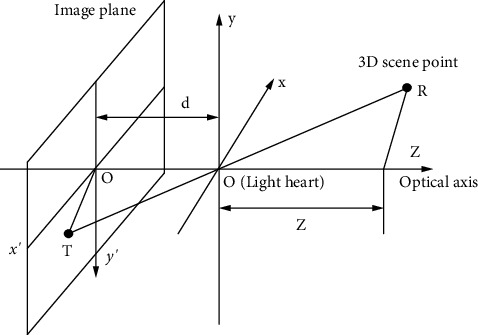
Coordinate system.

The image coordinate system generally performs image processing in the pixel coordinate system of the image arrangement to indicate the position of the image pixels in the image arrangement, as shown in Figure 2. The projection coordinates of the image plane may perform an image processing process by converting the image coordinate system in the imaging process [23].

If the same-order coordinates of a specific point *m* in the world coordinate system and the camera coordinate system are ${\left[\begin{array}{cccc} X & Y & Z & U \end{array}\right]}^{N}$ and ${\left[\begin{array}{cccc} x & y & z & u \end{array}\right]}^{N}$, respectively, the following relationship exists:

(8) $$\begin{array}{c} \left[\begin{array}{c} x\\ y\\ z\\ u \end{array}\right]=\left[\begin{array}{cc} M & n\\ O & u \end{array}\right]\left[\begin{array}{c} X\\ Y\\ Z\\ U \end{array}\right]\text{.} \end{array}$$

*M* is an orthogonal arrangement of 3^*∗*^3 units, and *n* is a 3^*∗*^1 translation vector of the image plane coordinate system.

The pixel coordinates of the computer image coordinate system are pixel units. Assuming that the origin of the image plane coordinate system is in the computer image coordinate system, the origin coordinates can be expressed as (*a*_0_, *b*_0_). In the computer image coordinate system, it is usually assumed that the physical size of each pixel of the *x*′-axis and the *y*′-axis is *fx*′, *fy*′. The relationship between the coordinates (*a*, *b*) of any pixel in the image of the computer image coordinate system and the corresponding coordinates *F* (*a*, *b*) in the image plane is as follows:

(9) $$\begin{array}{c} a=\frac{x^{\prime }}{fx^{\prime }}+a_{0}\text{,} \end{array}$$

(10) $$\begin{array}{c} b=\frac{y^{\prime }}{fy^{\prime }}+b_{0}\text{.} \end{array}$$

Using secondary coordinates, equations (9) and (10) can be expressed as follows:

(11) $$\begin{array}{c} \left[\begin{array}{c} a\\ b\\ u \end{array}\right]=\left[\begin{array}{ccc} \frac{1}{fx^{\prime }} & 0 & a_{o}\\ 0 & \frac{1}{fy^{\prime }} & b_{0}\\ 0 & 0 & u \end{array}\right]\left[\begin{array}{c} x^{\prime }\\ y^{\prime }\\ u \end{array}\right]\text{.} \end{array}$$

The inverse equation of (11) can be expressed as follows:

(12) $$\begin{array}{c} \left[\begin{array}{c} x^{\prime }\\ y^{\prime }\\ u \end{array}\right]=\left[\begin{array}{ccc} fx^{\prime } & 0 & -a_{o}fx^{\prime }\\ 0 & fy^{\prime } & -b_{0}fy^{\prime }\\ 0 & 0 & u \end{array}\right]\left[\begin{array}{c} a\\ b\\ u \end{array}\right]\text{.} \end{array}$$

#### 2.2.1. Imaging Model

The conversion between a three-dimensional scene and a two-dimensional scene is the most commonly used technology in imaging systems. In the process of converting three-dimensional images into two-dimensional images, most of them will choose to use perspective projection transformation and orthogonal projection transformation [24]. People tend to use perspective projection transformation rather than orthogonal projection transformation in real scenes.  Figure 1 shows a schematic diagram of perspective projection inverted imaging geometry.

**Figure 3:**
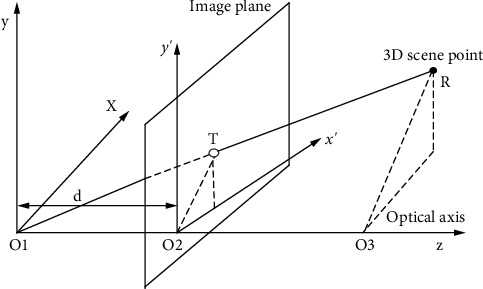
Optical flow and splicing.

As the most commonly used imaging model, perspective projection usually reverses the image when performing perspective projection. In order not to produce an inverted image during perspective projection, the image plane will be placed in front of the projection center most of the time.  Figure 3 shows a schematic diagram of perspective projection geometry.

**Figure 4:**
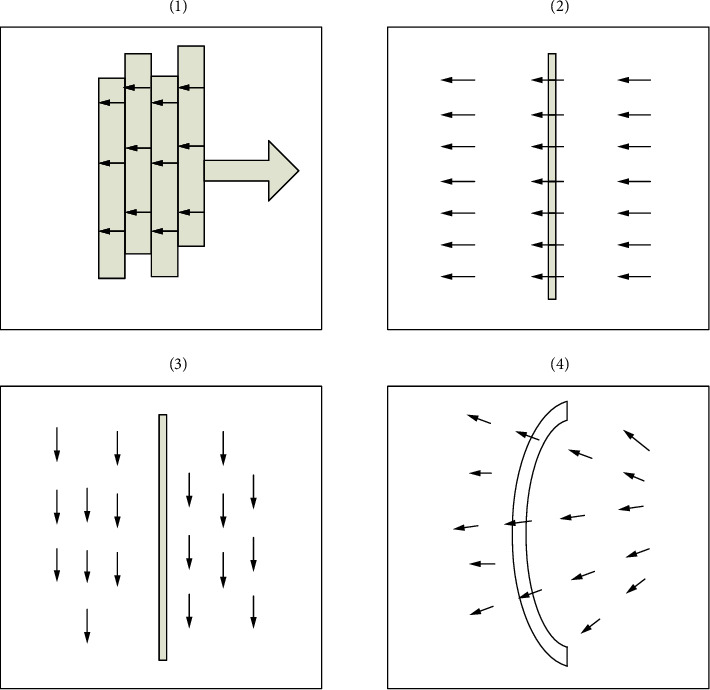
Schematic diagram of perspective projection geometry.

In Figure 4, the line of sight RO1 from the point *R* (*a*, *b*, *c*) of the 3D scene, the vertical line RO3 from the point *R* (*a*, *b*, *c*) to the *z*-axis, and O1R1 form a triangle. The line of sight TO3 of point *T* (*a*′, *b*′) on the image plane, the vertical line from point *T* (*a*′, *b*′) *D* to the *z*-axis, and the *z*-axis form a triangle. These two triangles are similar triangles, from which the perspective projection equation is obtained:

(13) $$\begin{array}{c} \frac{a^{\prime }}{a}=\frac{b^{\prime }}{b}=\frac{d}{c}\text{.} \end{array}$$

The position of the 3D scene point in the image plane is given by the following equation:

(14) $$\begin{array}{c} a^{\prime }=\frac{d}{c}a\text{,}\\ b^{\prime }=\frac{d}{c}b\text{.} \end{array}$$

In the formula, (*a*, *b*, *c*) represents the coordinates of the scene point *R* in the camera coordinate system, and (*a*′, *b*′) represents the coordinates of the *T* point in the plane image. The above-mentioned projection relationship can be expressed by a homogeneous coordinate equation, and the specific expression is as follows:

(15) $$\begin{array}{c} c\left[\begin{array}{c} a^{\prime }\\ b^{\prime }\\ u \end{array}\right]=\left[\begin{array}{cccc} d & 0 & 0 & 0\\ 0 & d & 0 & 0\\ 0 & 0 & u & 0 \end{array}\right]\left[\begin{array}{c} a\\ b\\ c\\ u \end{array}\right]\text{.} \end{array}$$

If equations (8) and (12) are substituted into (15), the relationship between the coordinates of the 3D scene point *R* represented by the world coordinate system and the coordinates of the projection point *T* in the computer image coordinate system can be obtained:

(16) $$\begin{array}{c} z\left[\begin{array}{c} a\\ b\\ u \end{array}\right]=\left[\begin{array}{ccc} \frac{1}{fx^{\prime }} & 0 & 0\\ 0 & \frac{1}{fy^{\prime }} & 0\\ 0 & 0 & 0 \end{array}\right]\left[\begin{array}{cccc} d & 0 & 0 & 0\\ 0 & d & 0 & 0\\ 0 & 0 & u & 0 \end{array}\right]\left[\begin{array}{cc} M & n\\ O & u \end{array}\right]\left[\begin{array}{c} X\\ Y\\ Z\\ U \end{array}\right]=E\left[\begin{array}{c} X\\ Y\\ Z\\ U \end{array}\right]\text{.} \end{array}$$

Among them, *E* is called the projection matrix.

#### 2.2.2. Mosaic of Endoscopic Images

Generally speaking, the splicing of endoscopic images mainly includes several steps of endoscopic image acquisition, image preprocessing, image registration, reprojection model selection, and image fusion [25].

*(1) Manifold Stitching*.  Figure 3 shows the general process of endoscopic image stitching.

If the displacement of two adjacent images is small, the two images can be regarded as the relationship of affine motion, so there is the following calculation formula:

(17) $$\begin{array}{c} \left[\begin{array}{c} a\\ b \end{array}\right]=\left[\begin{array}{c} x_{i}-x_{i-1}\\ y_{i}-y_{i-1} \end{array}\right]=\left[\begin{array}{c} m+nx_{i}+vy_{i}\\ z+ax_{i}+by_{i} \end{array}\right]\text{.} \end{array}$$

Then, the transformation relationship between image *T*_*i*−1_ and image *T*_*i*_ is

(18) $$\begin{array}{c} T_{i-1}=M_{i}T_{i}\text{,} \end{array}$$

where *M*_*i*_ is

(19) $$\begin{array}{c} M_{i}=\left[\begin{array}{ccc} 1-n & -v & -m\\ -a & 1-b & -z\\ 0 & 0 & 0 \end{array}\right]=\left[\begin{array}{ccc} \cos\;\theta *sx & -\sin\;\theta *shx & fx\\ \sin\;\theta *shy & \cos\;\theta *sy & fy\\ 0 & 0 & u \end{array}\right]\text{.} \end{array}$$

**Figure 5:**
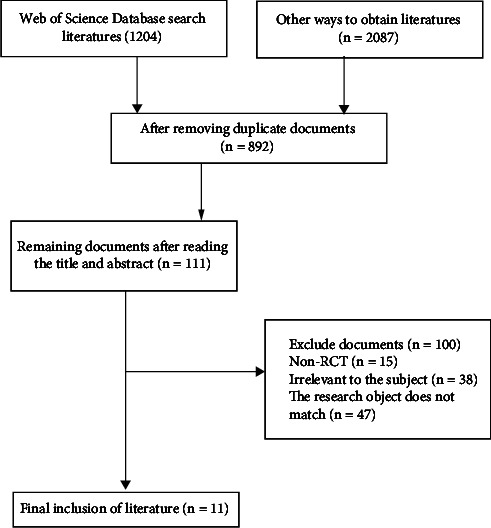
Document retrieval process.

According to Figure 5, to achieve cutting, it needs to find the line *X* (*x, y*) = 0 perpendicular to all optical flows. Since the normal vector of *X* (*x, y*) is perpendicular to *X*, we can get the following equation:

(20) $$\begin{array}{c} \left(\begin{array}{c} \frac{\partial M}{\partial x}\\ \frac{\partial M}{\partial y} \end{array}\right)=L\left(\begin{array}{c} a\\ b \end{array}\right)=L\left(\begin{array}{c} m+nx+vy\\ z+ax+by \end{array}\right)\text{.} \end{array}$$

When *v* = *a*, the solution of *M* can be obtained from equation (20) as follows:

(21) $$\begin{array}{c} 0=M\left(x,y\right)=mx+zy+\frac{n*x^{2}}{2}+\left(v+a\right)x*y+\frac{b*y^{2}}{2}+Y\text{.} \end{array}$$

## 3. Method

### 3.1. Search Strategy

The computer searches the web of science and CNKI. The search period is from the establishment of the database to August 2022. The search adopts the combination of free words and subject words and uses the literature tracking method to find relevant literature. The search terms in the English database include “mesocolon excision” and “laparoscopy”; The search terms in the Chinese database include “mesocolon excision,” “laparotomy” and “laparoscopy.”

### 3.2. Literature Inclusion and Exclusion Criteria

The inclusion criteria are as follows: ① the study design type is a randomized controlled trial or quasirandomized controlled trial. ② The study subjects in the literature were patients with mesocolon excision. ③ The surgical methods used by the experimental group in the literature include laparoscopic assisted treatment and Da Vinci intelligent robot assisted ④ the outcome indicators include the number and situation of postoperative complications.

Exclusion criteria are as follows: ① documents without full text, incomplete information or unable to extract data. ② Duplicate publication. ③ Minutes of meeting.

### 3.3. Literature Screening and Data Extraction

Two reviewers independently screened the preliminary literature strictly according to the inclusion and exclusion criteria. After excluding the trials that obviously did not meet the inclusion criteria, the full text of the potentially relevant literature was analyzed. The latter two reviewers cross-checked the results of the included studies and decided on the divergent studies through discussion or with the third investigator. For those with incomplete reports of included literature information, they can be supplemented by contacting the authors.

### 3.4. Meta-Analysis

This study used RevMan5.3 software for meta-analysis. Meta-studies generally have heterogeneity, which can be divided into methodological heterogeneity, clinical heterogeneity, and statistical heterogeneity. The heterogeneity of methods is caused by differences in research design and quality. Clinical heterogeneity is caused by differences in participants, interventions, and endpoint indicators. Statistical heterogeneity is based on data and overlaps with the confidence interval between studies. Correlation, the degree of coincidence is large, and the heterogeneity is small, on the contrary, the heterogeneity is large. If meta-analysis includes studies with large heterogeneity, it will reduce the credibility of the conclusions. At the same time, the existence of heterogeneity among various studies determines the choice of the combined effect size model.

## 4. Results

### 4.1. Literature Search Results

The preliminary inspection obtained 3291 documents. After reading the title and abstract, 2399 articles whose subjects and interventions did not meet the inclusion criteria of this study were excluded, and 111 papers that might meet the inclusion criteria were obtained through preliminary screening. After reading the full text and tracing the references of the included documents, no other documents related to this study were found. Further reading was screened according to inclusion and exclusion criteria and data integrity. 100 articles were excluded and 11 articles were included for meta-analysis. The document retrieval process is shown in Figure 5.

### 4.2. Quality of Included Studies

The methodological quality evaluations of the included 11 studies were all *B*-level.

### 4.3. Meta Analysis Results

This study analyzed the application effect of laparoscopy in mesocolon excision and the postoperative complications of patients with two surgical methods.

### 4.4. Theoretical Knowledge

Eleven studies evaluated the application effect of virtual reality technology at the theoretical knowledge level. The results showed that the heterogeneity of the included studies was low (*P* = 0.21, *I*2 = 24%), so the fixed effect model was adopted. The comprehensive results showed that compared with open surgery, the use of laparoscopy can effectively reduce the probability of postoperative complications [*Z* = 5.02, 95% CI (0.37, 0.65), *P* < 0.01].

### 4.5. Satisfaction Survey

The evaluation process of surgical effect is based on certain clinical indicators or the quality of life of patients after surgery. The occurrence of postoperative complications can effectively judge the quality of surgery. We used meta-analysis to make statistics on the literature used in this article. The results are shown in Table 1.

#### 4.5.1. Analysis of Experimental Results

**Figure 6:**
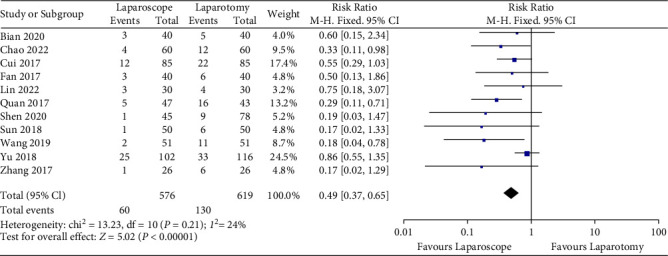
Meta-analysis forest plots expressed in ≤60 years old and >60 years old.

A meta-analysis of 11 articles was performed, showing laparoscopic and open surgery in 90 patients, respectively, as shown in Figure 6.

**Figure 7:**
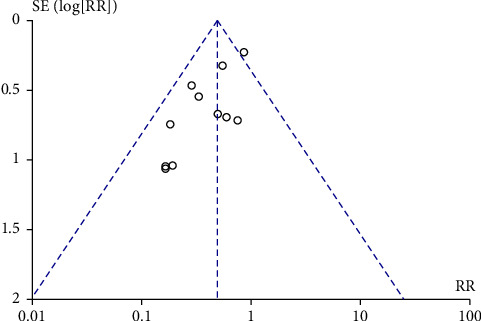
Funnel plot of comparison: 1 laparoscope vs. laparotomy.

Figure 7 shows the funnel chart results of 11 meta-analyses. The funnel chart results show that the distribution of the literature in this study is balanced and the reliability of the results is high.

## 5. Discussion and Conclusion

### 5.1. Advantages of Laparoscopic Surgery

Through meta-analysis, this study concluded that laparoscopy and electronic imaging technology can be fully combined with surgical clinical treatment, which can effectively improve the quality of life of patients. Modern clinical treatment should pay attention to the development and application of medical imaging technology, especially the diagnosis and treatment of complex surgery. Therefore, the clinical medical college should increase the investment in emerging technologies. The organic combination of electronic imaging technology and clinical practice can play an important role in the future clinical surgery practice.

### 5.2. Influence of Laparoscopic Treatment on Clinical Operation Effect

Generally speaking, electronic intelligent imaging technology is currently a relatively active technology, and clinical practice makes the future development of this technology full of vitality. Although significant results have been achieved in the practical application, there is still a more general gap between the requirements of clinical application. The development of this technology in China is relatively late, but with the gradual attention of relevant departments to virtual reality technology, the development of the Da Vinci surgical robot will be more prominent in clinical medicine, which will bring new development opportunities for future clinical treatment. At the same time, this meta-analysis has the following limitations: the number of studies included is small and the sample size is small. The results of the meta-analysis still need to be further verified by more large sample multicenter studies.

## Figures and Tables

**Table 1:** Main features of the study.

Author (Year)/Country	Design	Type of study	Number of patientsin the laparoscopicgroup	Number of patientsin the open surgerygroup	Bias riskgrade
Bian (2020)/China	RCT	Randomized trial	40	40	*B*
Chao (2022)/China	RCT	Randomized trial	60	60	*B*
Cui (2017)/China	RCT	Randomized trial	85	85	*B*
Fan (2017)/China	RCT	Randomized trial	40	40	*B*
Lin (2022)/China	RCT	Randomized trial	30	30	*Be*
Quan (2017)/China	RCT	Randomized trial	47	43	*B*
Shen (2020)/China	RCT	Randomized trial	45	78	*B*
Sun (2018)/China	RCT	Randomized trial	50	50	*B*
Wang (2019)/China	RCT	Randomized trial	51	51	*B*
Yu (2018)/China	RCT	Randomized trial	102	116	*B*
Zhang (2017)/China	2-Arm RCT	Randomized trial	26	26	*B*

## Data Availability

The data used to support the study are included in the paper.
